# Evaluating architecture impact on system energy efficiency

**DOI:** 10.1371/journal.pone.0188428

**Published:** 2017-11-21

**Authors:** Shijie Yu, Hailong Yang, Rui Wang, Zhongzhi Luan, Depei Qian

**Affiliations:** 1 Sino-German Joint Software Institute, School of Computer Science and Engineering, Beihang University, Beijing, China; 2 School of Data and Computer Science, Sun Yat-sen University, Guangzhou, China; Chongqing University, CHINA

## Abstract

As the energy consumption has been surging in an unsustainable way, it is important to understand the impact of existing architecture designs from energy efficiency perspective, which is especially valuable for High Performance Computing (HPC) and datacenter environment hosting tens of thousands of servers. One obstacle hindering the advance of comprehensive evaluation on energy efficiency is the deficient power measuring approach. Most of the energy study relies on either external power meters or power models, both of these two methods contain intrinsic drawbacks in their practical adoption and measuring accuracy. Fortunately, the advent of Intel Running Average Power Limit (RAPL) interfaces has promoted the power measurement ability into next level, with higher accuracy and finer time resolution. Therefore, we argue it is the exact time to conduct an in-depth evaluation of the existing architecture designs to understand their impact on system energy efficiency. In this paper, we leverage representative benchmark suites including serial and parallel workloads from diverse domains to evaluate the architecture features such as Non Uniform Memory Access (NUMA), Simultaneous Multithreading (SMT) and Turbo Boost. The energy is tracked at subcomponent level such as Central Processing Unit (CPU) cores, uncore components and Dynamic Random-Access Memory (DRAM) through exploiting the power measurement ability exposed by RAPL. The experiments reveal non-intuitive results: *1)* the mismatch between local compute and remote memory node caused by NUMA effect not only generates dramatic power and energy surge but also deteriorates the energy efficiency significantly; *2)* for multithreaded application such as the Princeton Application Repository for Shared-Memory Computers (PARSEC), most of the workloads benefit a notable increase of energy efficiency using SMT, with more than 40% decline in average power consumption; *3)* Turbo Boost is effective to accelerate the workload execution and further preserve the energy, however it may not be applicable on system with tight power budget.

## Introduction

For decades, the advancements in computer architecture are undoubtedly pushing the frontier of system performance, fulfilling the prophecy of Moore’s Law. However, it becomes well accepted nowadays that the computer systems can not continue to reap the benefits of the existing architecture designs without considering energy efficiency [1]. Especially, for large scale computer systems that are pervasively deployed in HPC and datacenter environment hosting tens of thousands of interconnected computers, energy consumption is no longer a second class citizen but becomes a major concern in daily operation. Similarly, the energy consumption has already been payed significant attention in other research areas such as vehicle electronics and electrification [2–5]. The shifting emphasis promotes the system developers to re-evaluate the architecture designs in terms of energy efficiency other than raw performance. Although some of the hardware designs have been existing for several decades and tremendous efforts have been devoted to understand their performance capability, comprehensive study on how these architecture designs affect system energy consumption is missing, and thus hinders intelligent strategies to be applied optimizing system energy efficiency.

Energy proportionality [6] is an attractive merit for future computer hardwares. Theoretically, system built with energy proportional hardwares consumes energy strictly adhere to its actual resource usage with no additional cost. For instance, no energy should be consumed as long as the system remains idle. However, the fulfillment of energy proportionality requires fundamental breakthroughs in material science as well as to overcome the formidable manufacturing obstacles. Therefore, there is still a long way for its wide adoption in real systems. Even though energy proportionality is possible in the near future, the diversity of application characteristics, interacting software layers and distinct system configurations are all factors to prevent the system reaching its peak performance and thus offsetting the benefits provided by energy proportional hardwares. It is imperative to design and implement adaptive strategies based on a thorough evaluation of the existing architecture designs, especially from the energy perspective, in order to bridge the energy proportionality gap in foreseeable computer systems.

The ability of power measurement plays an important role in energy study, since measurement at fine granularity as well as with high accuracy can reveal more details about system behaviors on energy consumption. Previous work [7–10] either relies on external power meters or power models to obtain the system energy consumption. However, each approach has its own strength and weakness in practice. For the power meter approach [7, 8], although it is competent in fine granularity and high accuracy, it requires additional devices to be purchased with extra financial cost which prohibits its wide adoption in large scale environment. Whereas, the power model approach [9, 10] is pure software based utilizing statistical methods to correlate the energy consumption with resource usage. The drawback of the modeling approach is that most of the power models are with limited accuracy, since they are not exposed to the energy specification at the level of transistors and circuits of the underlying hardwares. Therefore, we argue the existing power measurement approaches are either impractical or inaccurate to evaluate the impact of different architecture designs on system energy consumption especially at subcomponent granularity.

The advent of Intel Running Average Power Limit (RAPL) interfaces [11] combines the advantage of both hardware and software measurement approaches. The RAPL interfaces leverage built-in power sensors collecting voltage and current ranging from CPU, Last Level Cache (LLC), bus interconnect to DRAM, as well as sophisticated power models to predict the energy consumption on different system components instantaneously. The power measurement ability exposed by RAPL enables measuring the system energy consumption at fine granularity (approximately 1 millisecond interval) on multiple system components that was impossible before, which provides an unique opportunity to reason about how architecture designs affect the system energy consumption in unprecedented details. In the meanwhile, we expect the in-depth architecture evaluation reveals non-intuitive insights to guide system and application optimization exploiting the strength of different architecture designs toward better energy efficiency.

The goal of this study lies on investigating the impact of the existing architecture designs including NUMA, SMT and Turbo Boost, in terms of energy efficiency. We leverage representative benchmark suites and the RAPL interfaces to provide fine-grained energy measurement. Specifically, this paper makes the following contributions:

- We identify the energy proportionality gap on real system via quantitive analysis of the power consumption on different system components.
- The deviation on the time interval for the RAPL interfaces to update the energy registers is studied, which determines whether it is necessary to align the energy measurement with the RAPL updates regarding the specified measurement granularity.
- A comprehensive evaluation of power consumption on each system components and system level energy efficiency is presented with the NUMA, SMT and Turbo Boost enabled respectively. In addition, detailed interpretation of the architecture strength considering the diverse characteristics of the representative benchmark suites is complemented based on experiment observations.

The remainder of the paper is organized as follows. Section 1 highlights the uniqueness of our work by discussing the related work. Section 1 illustrates the energy proportionality gap with quantitive analysis and describes the capability of RAPL power measurement. Section 1 introduces the methodology applied to evaluate the architecture designs, including the considerations to choose representative benchmark suites and identify the deviation of RAPL energy update interval. Section 1 presents the detailed analysis results to reveal the energy impact of different architecture designs. Finally, section 1 presents our conclusion and future work.

## Related work

Prior research works on computer architecture have primarily concentrated on accelerating system performance. Sato et al. [12] argue the importance of single core performance even in the multicore era, and propose a technique to improve single core performance based on Intel Turbo Boost. Majo et al. [13] design and implement a small set of language-level primitives for memory allocation and loop scheduling to eliminate the costly remote memory access caused by the NUMA architecture. Su et al. [14] present algorithms and a runtime system that optimize the execution of Open Multi-Processing (OpenMP) applications through thread placement to minimize the critical path of OpenMP parallel regions on NUMA architectures. Ramirez et al. [15] illustrate RaT, an interesting design choice for SMT processor that would influence the way in which future SMT processors balance resource usage between ILP and memory-bound threads. Hiroshi Inoue and Toshio Nakatani [16] explore the performance between multi-process and multi-thread processing on a multi-core SMT processor. Their evaluation shows that both models achieve almost comparable core scalability, whereas the multi-thread model achieves much better SMT scalability and higher performance. DeVuyst et al. [17] propose scheduling policies on chip multiprocessors with simultaneous multithreading cores, which allows the system to identify and migrate threads toward better performance and energy efficiency. However, none of these research works can provide deep insights in understanding the role of the existing architecture designs from the energy efficiency perspective.

At the same time, tremendous efforts have been devoted to improve the power measurement methodology. [18, 19] conclude that RAPL offers a viable alternative to physical power meters while the only drawback is lack of interfaces that would allow measuring the energy consumption of the main memory as RAPL only offers counters related to the operation of the main memory controller. Khanna et al. [20] develop UEFI based firmware methodology to allow energy aware memory allocation, accurate DRAM energy measurement and efficient energy limiting. The methodology optimizes the DRAM locality as well as enhances the accuracy of RAPL. Weaver et al. [21] propose the PAPI performance analysis library to measure energy and power consumption of the workload incorporating the capability of RAPL. Rountree et al. [22] quantify the power envelope on Xeon Sandy Bridge server. They also explore the potential of RAPL as a DVFS replacement and explain how the RAPL technology can measure and limit power. Distinct from the above work, our study leverages the superior power measurement capability of RAPL to evaluate the energy impact of architecture designs such as NUMA, SMT and Turbo Boost.

There are emerging research works trying to understand the energy consumption from both hardware and software aspects. Subramaniam et al. [23] investigate the possibility to achieve energy proportionality for an enterprise-class server workload with RAPL power limiting ability. Chandrasekar et al. [24] demonstrate the effects of process variations on DRAM performance and power consumption with over-estimation up to 28% by the vendor. Balaji et al. [25] characterize the variability in power consumption of modern mobile processors, and the fine-grained power measurements reveal the variability across parts is indeed significant, ranging from 7% to 17%. Esmaeilzadeh et al. [26] quantitatively analyze the measured power and performance at the chip level across five hardware generations using diverse benchmarks. Their results suggest it is necessary to expose on-chip power meters to the researchers in order to optimize the power and performance. Schone et al. [27] evaluate the energy efficiency of SMT on on state-of-the-art x86_64 processors. Hahnel et al. [28] leverage RAPL to measure the energy cost of short code path for decoding video slices. Despite the pioneering explorations in reasoning the energy consumption, a comprehensive evaluation of the existing architecture designs on their impact to energy efficiency is missing. Our study complement this absence through characterizing the energy behavior of representative benchmark suites at subcomponent level with fine-grained time resolution and high accuracy.

In addition to the effort on general purpose hardware, researchers are resorting to special hardwares for energy efficiency in the era of dark silicon [29]. Especially due to the recent success of deep neural networks, large body of research works [30–34] have been devoted to design energy efficient hardwares for Deep Neural Networks (DNNs). The Diannao family [30] propose a series of hardware accelerators specially designed for neural networks with an emphasis on the impact of performance as well as energy efficiency. [31] elaborate the effort at Microsoft to implement a Convolution Neural Network (CNN) accelerator using server augmented with Field Programmable Gate Arrays (FPGAs). Their evaluation results show promise performance comparable to high-end Graphic Processing Unit (GPU) but only a fraction of its power consumption. Moreover, several optimizations are proposed to further improve the energy efficiency of DNN accelerators from both algorithm [32] and data movement [33] perspectives. Minerva [34] presents a highly automated co-design approach across the algorithm, architecture, and circuit levels to optimize DNN hardware accelerators. These studies are orthogonal to our energy characterization on general purpose processor. In addition, the characterization methods in our paper can also be applied to special hardware to understand the energy impact of different designs.

## Energy background

In this section, we first elaborate the discrepancy between current enterprise server and its energy proportional counterpart in ideal by running benchmark workload on real system. Then, we briefly introduce the power measurement capability provided by RAPL interfaces.

### Energy proportionality gap

The energy proportionality gap is commonly used to depict the difference of energy consumption between current deficient computer system from its appealing counterpart composed of energy proportional hardwares. In the definition of energy proportionality, the ideal computer system is supposed to consume energy proportional to its resource utilization, with no energy cost when the system is idle. In order to articulate the energy discrepancy of the real world system from the energy proportional expectation with quantitative data, we perform the workload Embarrassingly Parallel (*ep*) from the NAS Parallel Benchmarks-Message Passing Interface (NPB-MPI) benchmark suite with different input scales to observe the system energy behavior. The workload requires a significant amount of computational resources from the system, stressing the CPU component intensively. We execute *ep* with six MPI processes to fully occupy the physical cores on a state-of-the-art Intel processor with increasing input scales. The RAPL interfaces are utilized to measure the average power consumption on different system components.

**Fig 1:**
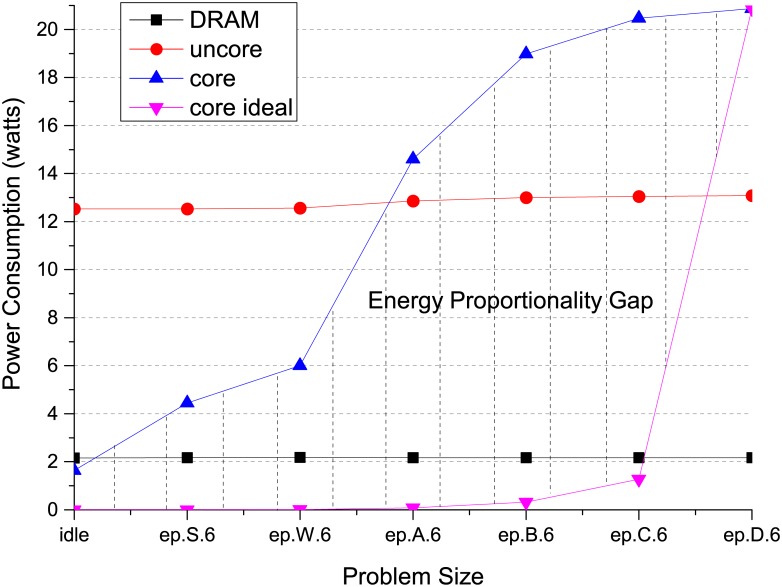
The energy proportionality gap for each system component with the *ep* workload running at different input scales.

As shown in Fig 1, the power consumption of DRAM remains almost constant under different load levels, indicating the DRAM is the most non energy proportional component in the system. It is similar with the uncore components that the power consumption stays stable regardless of the input scale, which includes hardwares such as LLC, snoop pipeline and memory controller. Even with the energy efficient component as CPU, it is far beyond the energy proportional behavior, which is identified in the shaded area in Fig 1. The ideal power consumption of energy proportional CPU is extrapolated by assuming the system is fully utilized with *ep* running at input scale *D*. According to the NPB input specification, the problem size of each input scale is shown in Table 1. The energy proportional CPU power consumption is derived by normalizing other input scale to input scale *D*, then multiplied with the maximum system power consumption. We believe the huge energy proportional gap will continue to exist for the coming decades. Therefore, it is necessary to leverage the architecture designs to bridge such gap based on thorough evaluation with representative benchmarks from energy efficiency perspective.

**Table 1:** The size of the problem at different input scale for workload *ep*.

Workload	Scale S	Scale W	Scale A	Scale B	Scale C	Scale D
EP	2^24^	2^25^	2^28^	2^30^	2^32^	2^36^

### Power measurement

As the ability to perform effective power management is highlighted on Intel’s 2nd and 3rd generation processor architectures, RAPL has played an important role in fulfilling the promise. RAPL is designed and implemented to support power management on critical system components with high resolution and accuracy. It relies on reading and writing a certain range of bits within the Machine Specific Registers (MSRs) to profile and control the system energy consumption. Instead of developing a specific kernel driver to manipulate MSRs, the MSRs have already been incorporated into the kernel modules, which are loaded automatically when the kernel is started.

In the view of RAPL, the processor socket is divided into three domains: *1)* Package (PKG), *2)* Power Plane 0 (PP0) and *3)* DRAM. The PKG domain measures the energy consumption of the whole CPU socket. In multi-socket system, after RAPL modules are loaded by the kernel, the RAPL interfaces are exported through */dev/cpu/X/msr* register file associated with each socket respectively. While the PP0 domain consists of all the CPU cores in the same socket exclusively, the DRAM domain monitors the power consumption of the memory node connected to the CPU socket. Note that there is also a PP1 domain that manages the energy consumption of the graphic cards, which only exists on the client machine with graphic card on chip. Another implicit domain worth to be mentioned is the uncore domain that covers the components critical to performance such as LLC, memory controller and snoop pipeline. The energy consumption of the uncore domain is usually derived by subtracting the energy profile of PP0 from PKG.

## Evaluation methodology

In this section, we first describe the benchmark suits selected to characterize the energy behaviors of the architecture designs. Then we verify the resolution for RAPL updating its internal energy registers, which is an indispensable property for fine grained energy measurement.

### Benchmark suites

A well-defined benchmark suite should contain relevant workloads that represent important applications in contemporary computing facilities such as HPC and datacenter, and be diverse enough to stress various aspects of the architecture designs. In this study, we select workloads exhibiting a broad spectrum of characteristics from the SPECCPU 2006v1.1 [35], PARSEC v2.1 [36] and NPB-MPI v3.3 [37], as listed in Table 2. SPECCPU 2006 is an industry standard and well accepted benchmark suite that is pervasively used in system performance evaluation. While PARSEC benchmark suite represents the emerging multi-core applications incorporating workloads from multiple domains, and NPB-MPI stands for the traditional HPC workloads derived from NASA real fluid computational applications.

**Table 2:** Benchmark suites of representative workloads.

Benchmark Suite	Parallelization	Workloads
NPB-MPI	MPI	MultiGrid (*mg*), Conjugate Gradient (*cg*), Fast Fourier Transform (*ft*), Integer Sort (*is*), Embarrassingly Parallel (*ep*), Block Tridiagonal (*bt*), Scalar Pentadiagonal (*sp*), Lower-Upper symmetric Gauss-Seidel (*lu*)
SPECCPU	-	*astar*, *bwaves*, *bzip2*, *cactusADM*, *calculix*, *dealII*, *gamess*, *gcc*, *GemsFDTD*, *gobmk*, *gromacs*, *h264ref*, *hmmer*, *lbm*, *leslie3d*, *libquantum*, *mcf*, *milc*, *namd*, *omnetpp*, *povray*, *sjeng*, *soplex*, *specrand*, *sphinx3*, *tonto*, *wrf*, *xalancbmk*, *zeusmp*
PARSEC	Pthread	*blackscholes*, *bodytrack*, *ferret*, *freqmine*, *raytrace*, *swaptions*, *vips*, *x264*

According to the characteristics of the workloads, the benchmark suites can be briefly divided into two categories, serial and parallel workloads. The serial benchmark SPECCPU is leveraged to evaluate the architecture impact of Turbo Boost, while the parallel benchmarks of NPB-MPI and PARSEC are used to stress the NUMA and SMT architecture designs respectively. Note that we are not using multiple instances of the same SPECCPU workload as parallel workload to fully occupy the CPU cores, in contrast to previous work [27]. Since we believe multiple instances of serial workloads should be treated as multiple applications, which may cause severe interference to the analysis of architecture impact on energy consumption due to the unexpected contention on shared resources such as LLC and memory controller [38].

### RAPL measurement resolution

The Intel Developer Manual describes the fine grained energy measurement capability of RAPL at approximately 1 ms granularity, however, indicates the actual time resolution for RAPL to update the MSR registers varies from product to product without mentioning how large the deviation is. In some cases, such deviation may become unacceptable such as energy measurement at function and system call level. Therefore, it is important to identify the disparity between theoretical and actual measurement resolution for RAPL interfaces. In order to capture the RAPL update interval, we utilize an infinite loop to profile the RAPL interfaces continuously. Whenever there is a difference between current and previous energy reading, the time duration from last energy update is recorded into a log file for future analysis. As many as 5,000 samples are collected before we stop profiling. Since RAPL directly measures energy consumption of the system components, the energy readings increase monotonically. Thus it is valid for our method counting the energy difference to reveal the update interval.

**Fig 2:**
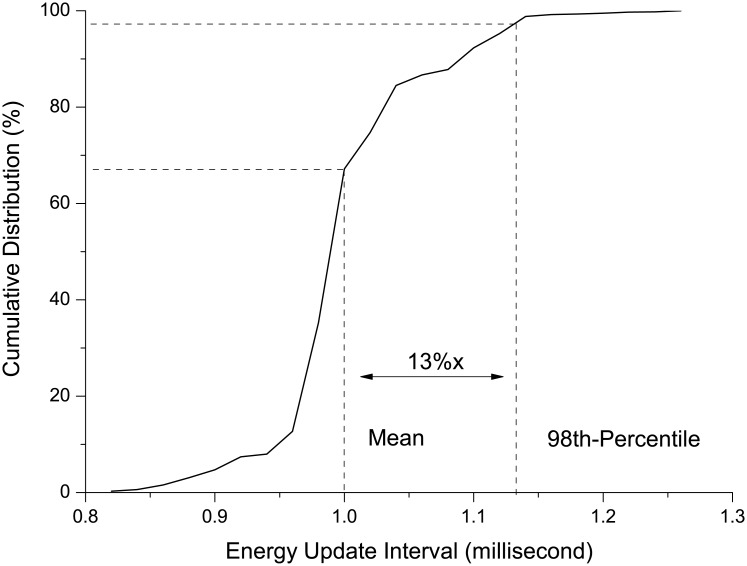
The cumulative distribution of the time interval for RAPL updating the energy registers. 98% of energy update interval fall in the range that less than 1.15 ms.

The profiling result is shown in Fig 2. The average time interval between two consecutive RAPL energy updates lies exactly in 1 ms. However, the margin between the mean and 98th percentile update interval is as large as 13%. The deviation between each RAPL energy update indicates additional alignment may be required depending on the specific energy measurement scenario. For instance, in our study the execution time of all the workloads within the benchmark suites is at second granularity. Thus the deviation less than one millisecond does not deteriorate the measurement accuracy. Whereas, for much finer grained energy measurement such as at function and system call level, delicate mechanism to align the energy measurement with the workload execution is necessary since the deviation significantly affects the measurement accuracy.

## Analysis of architecture impact

In this section, the hardware and software configurations applied in our evaluation are presented. Then the experimental results on energy efficiency when NUMA, SMT and Turbo Boost enabled respectively are elaborated with comprehensive analysis.

### Experimental setup

The experiments have been performed on two Intel Sandy Bridge servers with system configurations depicted in Table 3. The particular parameters to specify the range and granularity of the energy measurement supported by RAPL are defined in the *MSR_PKG_POWER_INFO*, *MSR_DRAM_POWER_INFO* and *MSR_RAPL_POWER_UNIT* registers. The range parameters of RAPL interfaces on the two systems are identical as shown in Table 4, with the energy unit 1.52 × 10^−6^ J. To note that, RAPL does not provide the power measurement ability directly instead of energy. In order to derive the average power consumption of each workload, the total execution time is recorded and then divided by the energy consumption.

**Table 3:** System configurations of two Sandy Bridge servers.

	**Server T1**
Vendor/Model	Intel Sandy Bridge EP
CPU Sockets	2x Intel Xeon E5-2620
Core per Socket	6
SMT	12 logical threads when enabled
Turbo Boost	2.0GHz(2.5GHz)
Memory	4x 4GB SamSung DDR3-1333
Motherboard	Lenovo RD630
Disk	3x 300GB SATA Seagate ST9300605SS
OS	CentOS 6.2
Linux Kernel	2.6.32-220.el6.x86_64
	**Server T2**
Vendor/Model	Intel Sandy Bridge EP
CPU Sockets	2x Intel Xeon E5-2609
Core per Socket	4
SMT	not supported
Turbo Boost	not supported
Memory	4x 8GB Kingston DDR3-1066
Motherboard	Supermicro X9DRG-QF
Disk	2x 128GB SATA Western Digital WD1003FBYX-01Y7B1
OS	CentOS 6.3
Linux Kernel	2.6.32-279.el6.x86_64

**Table 4:** Parameters of RAPL measurement range including Maximum Time Window (MTW), Maximum Power (MaxP) and Minimum Power (MinP).

Domain/Range	MTW	MaxP	MinP
PKG	46ms	150w	63w
DRAM	39ms	75w	15w

Most of the NPB-MPI workloads require the number of CPU cores to be power of two as well as N-th root. Therefore, we execute the NPB-MPI workloads on server T2 to evaluate NUMA impact, and other workloads on server T1 since SMT and Turbo Boost are not supported on server T2. In the meanwhile, one architecture feature is evaluated at a time with the rest disabled to eliminate the interference. We leverage the Linux command *numactl* to pin the threads/processes to one CPU node and remote memory node in the NUMA experiments, while in other experiments *taskset* is used to bind the threads/processes to particular CPU cores. The Instruction Per Cycle (IPC) is used as an indicator for workload performance.

### Non Uniform Memory Access

In order to measure the NUMA energy effect, we artificially constrain the memory accesses on socket 0 not to utilize its local memory node, instead they are redirected to the memory node adjacent to socket 1 through the Intel QuickPath Interconnect (QPI), so that the computation of the workload is performed locally with its memory space allocated remotely. To make fair comparison, the additional energy introduced by NUMA on the remote socket is also counted into the energy measurement.

**Fig 3:**
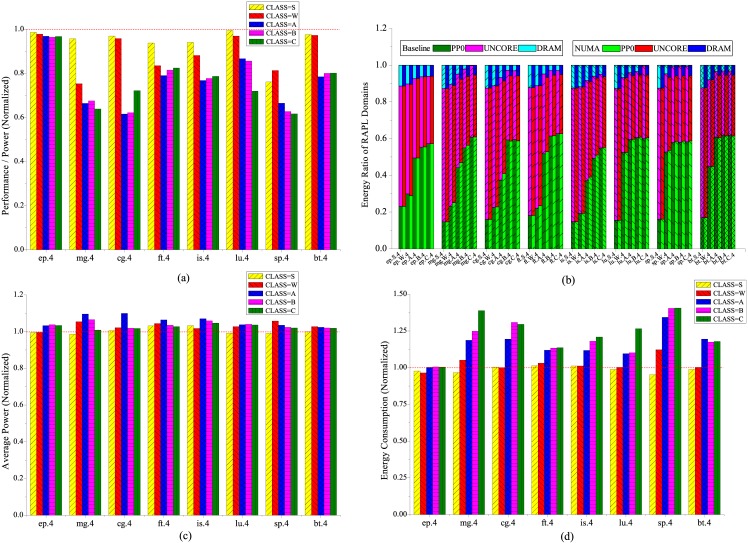
Energy characterization of NUMA with NPB workloads, (a) performance per watts (b) energy ratio of RAPL domains, (c) average power consumption and (d) total energy consumption. The results are normalized to NUMA disabled.

The X axis in Fig 3(a), 3(c) and 3(d) indicates the name of the workload as well as the number of MPI processes to run the workload. For instance, *ep.4* means we run the workload *ep* with four MPI processes. Whereas the X axis in Fig 3(b) includes more information such as the input scale. For instance, *ep.S.4* means we run the workload *ep* with four MPI processes at input scale *S*. Fig 3(a) depicts all the NPB-MPI workloads suffer from different amount of energy efficiency degradation when NUMA takes into effect. The workload *cg* experiences the most severe energy efficiency decline of more than 38.4% at input scale *A*, while the NUMA hardly causes significant impact on workload *ep* with energy efficiency decline less than 4% at all input scales. The difference in energy efficiency between these two workloads can be explained that *ep* is pure computation intensive with trivial memory accesses, thus the mismatch between CPU and memory through NUMA setting can not generate obvious influence on the execution of workload *ep*. Whereas workload *cg* shows large memory footprint, and the additional ad-hoc through QPI to the remote memory node not only prolongs the execution, but also introduces extra energy consumption on the remote socket. It is also notable that most of the workloads exhibits a large energy efficiency drop when the input scale goes beyond *W* except *ep*, however, there is a light increase of energy efficiency at the largest two input scales *B* and *C* with workloads *ft*, *is* and *bt*.

The energy ratio of the CPU cores increases uniformly for all workloads as the input scales, with the largest increase occurs at workload *mg* by from 14.7% (CLASS = S, NUMA) to 60.9% (CLASS = C, NUMA) when NUMA takes into effect as shown in Fig 3(b). Correspondingly, the energy ratio of DRAM becomes smaller as input scales due to more energy is consumed by the CPU cores. We also notice that workload *ep* is insensitive to the NUMA effect since the energy ratio of DRAM is almost the same compared to the baseline at all input scales. Whereas for the rest of the workloads, the energy ratio of DRAM increases apparently compared to the baseline when the input scales beyond *W*. The largest increase of DRAM energy ratio occurs at workload *sp* with input scale *B*, from almost 0.6% at baseline to 5.8% due to NUMA effect. The distinct DRAM energy sensitivity between workload *ep* and the others can be explained as follows. For workload *ep*, it is highly computation intensive with limited memory accesses. Thus the memory accesses to the remote NUMA node have almost no effect on the DRAM energy consumption of workload *ep* compared to the baseline. However, for the rest of the workloads, the number of memory accesses increases dramatically as the size of the input scales. The increased memory access latency due to NUMA leads to more energy consumed at the DRAM. As depicted in Fig 3(c) and 3(d), the power consumption for most of the workloads rises up less than 7% except that *mg* and *cg* at input scale *A* approach 10%, whereas the energy consumption has been elevated remarkably with maximum increase of workload *sp* at input scale *C* by more than 40.5%. The discordance of increase between power and energy indicates the remote memory accesses caused by NUMA generate dramatic latency overhead and prolongs the execution, which in turn accounts for additional energy consumption. During the experiments, we also notice that there is an obvious power increase on the remote compute and memory node by 8% on average, which can be attributed to the remote memory access that activates the LLC, memory controller and DRAM, preventing the CPU package to switch to deep sleep state.

**Insight-1 (NUMA)** *The energy efficiency of workload with intensive memory operations significantly deteriorates due to the mismatch of computation and memory accesses caused by NUMA, which not only prolongs the workload execution but also introduces extra energy consumed on other CPU sockets. This insight is quite important to design effective process/thread schedulers for load balance. Current load balance schedulers only take into account the CPU utilization, neglecting the impact of remote memory access due to NUMA. Therefore, when balancing the load, only the computation is migrated without carrying along its data. It is highly possible after migration the process/thread still needs to access its data residing on the remote memory node, which degrades both application performance and system energy efficiency. We recommend future load balance scheduler should be NUMA aware and take care of the data when performing process/thread migration.*

### Simultaneous multithreading

**Fig 4:**
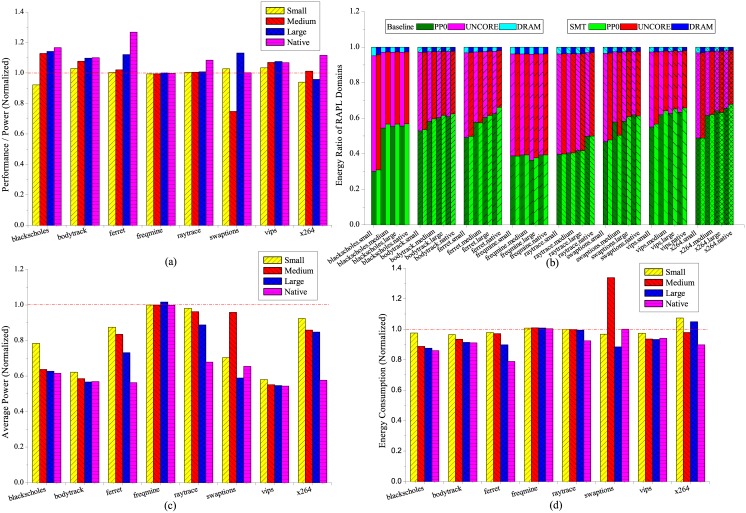
Energy characterization of SMT with PARSEC workloads, (a) performance per watts (b) energy ratio of RAPL domains, (c) average power consumption and (d) total energy consumption. The results are normalized to SMT disabled.

PARSEC workloads are utilized to evaluate the energy impact of SMT. The X axis in Fig 4(a), 4(c) and 4(d) indicates the name of the workload. Whereas the X axis in Fig 4(b) includes more information such as the input scale. For instance, *blackscholes.small* means we run the workload *blackscholes* at input scale *small*. As shown in Fig 4(a), the energy efficiency of all PARSEC workloads except *freqmine* has been improved at certain input scale when SMT is enabled, with the highest energy efficiency increase of 26.8% achieved by *ferret* at input scale *native*. The exception demonstrates the SMT is not beneficial to workload *freqmine* when its input scales. In contrast, we notice that some workloads maintains good scalability with SMT such as *blackscholes*, *bodytrack* and *ferret*. Compared to the rest of the workload execution, there is an abnormal behavior of workload *swaptions* at input scale *medium* with degrading energy efficiency of 25.2%.

The energy consumption ratio of different system components is depicted with RAPL domains in Fig 4(b). The DRAM consumes the least energy for all the workloads with average 2.9% of the total. On the contrary, the CPU cores are the most power hungry components when the input scales beyond *small*, taking up more than 50% of the entire system energy consumption except *freqmine* and *raytrace*, for which the uncore components such as LLC and memory controller dominate the energy consumption. As the input scales, the portion of CPU energy consumption increases obviously with most of the workloads. For workload *blackscholes*, the energy increase on CPU occurs immediately from input *small* to *medium* and remains constant for the rest of input scales, which also reaches the highest increase of 25.9% among other workloads. Whereas for other workloads such as *bodytrack*, *ferret*, *vips* and *x264*, the energy growth happens gradually in response to the input scale. In addition, there is a notable energy increase on CPU when SMT is enabled for most workloads except *freqmine* and *raytrace*. We also notice that with SMT enabled, the energy ratio of the CPU component increases slightly for workload *blackscholes*, *bodytrack* and *vips* at all input scales compared to the baseline. Whereas for workload *raytrace*, the energy ratio of different components remains the almost same compared to the baseline, and thus is insensitive to SMT. The amount of increase on the CPU energy ratio actually reflects how much the workload can explore the simultaneous processing from SMT. The larger increase on the CPU energy ratio, the higher performance the workload can achieve with SMT enabled.

It is demonstrated in Fig 4(c) that SMT is able to reduce the average power consumption significantly for most of the workloads when enabled. For the best case with workload *vips* at input scale *native*, the power saving reaches as much as 45.6%. However, the actual energy saving is not as perfect as power compared to Fig 4(d). For workloads except *freqmine*, *swaptions* and *x264*, the average energy saving is 4.2% at all input scales when SMT enabled. The tendency exhibited by *ferret* is more promising that the energy saving is in reverse proportion to input scales with 21.1% reduction in energy consumption at input scale *native*. The disparity between power and energy consumption of PARSEC workloads can be attributed to the prolonged execution due to shared resource contention such as LLC, memory controller and even arithmetic units.

**Insight-2 (SMT)** *In general, SMT is helpful to bring down the power and energy consumption for multithreaded applications, and effective to improve the energy efficiency for applications in this category. However in certain cases with SMT enabled, there is high possibility to generate severe contention on shared resources such as LLC, memory controller and arithmetic units. Especially for memory intensive applications (e.g., raytrace), on one hand with more SMT cores it consumes less power during the computation, on the other hand the interference due to shared resource contention apparently degrades the performance of the application, and thus offsets the benefit of less power consumption in the view of energy efficiency. Nevertheless, it is always recommended to turn on SMT when running applications in power constrained scenarios such as embedded and mobile system since it is quite effective to reduce the power consumption.*

### Turbo Boost

The SPECCPU is an appropriate benchmark candidate to evaluate the impact of Turbo Boost, since most of the workloads within SPECCPU are sensitive to CPU frequency. For our system on Server T1, there is only one Turbo step supported. Whenever the Turbo Boost is enabled, the CPU frequency jumps from 2.0 GHz to 2.5 GHz immediately. Each SPECCPU workload is constrained to execute on the first core of socket 0.

**Fig 5:**
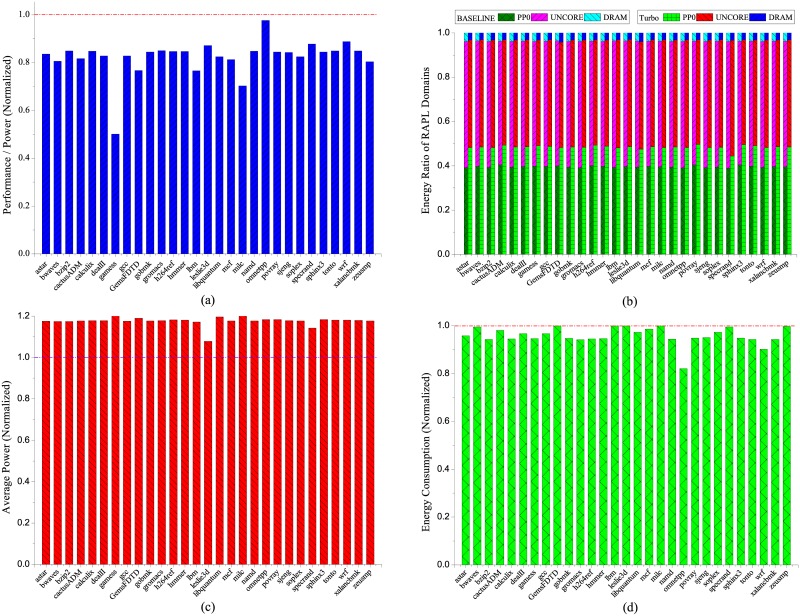
Energy characterization of Turbo Boost with SPECCPU workloads, (a) performance per watts (b) energy ratio of RAPL domains, (c) average power consumption and (d) total energy consumption. The results are normalized to Turbo Boost disabled.

The X axis in Fig 5 indicates the name of the workload. The results shown in Fig 5(a) is quite non-intuitive that the energy efficiency for most of the SPECCPU workloads uniformly decreases significantly with 17.6% on average. The best and worst case is achieved by workload *omnetpp* and *gamess* that the energy efficiency deteriorates by 2.4% and 49.9% respectively. The reason is explained later in this section. Fig 5(b) indicates the energy ratio of CPU cores is the most sensitive component to Turbo Boost, which increases uniformly across all the workloads compared to the baseline, with minimum increase of 5.4% (*specrand*) and maximum increase of 9.1% (*povray*). The uniform increase of the CPU energy ratio can be attributed to the increased frequency of CPU cores when Turbo Boost is enabled, which leads to larger portion of the energy consumption with CPU compared to other components.

Another interesting phenomenon revealed from Fig 5(c) and 5(d) is that although the average power consumption for most of the workloads has been raised up by more than 20%, the energy consumption is not actually increasing, some even reduced by as much as 17.9% (*omnetpp*). The opposite tendency of power and energy consumption indicates the execution of SPECCPU workloads can be significantly accelerated with Turbo Boost enabled, which offsets the increase in power consumption. However, memory intensive workloads such as *bwaves*, *GemsFDTD*, *lbm*, *leslie3d* and etc. hardly reap notable energy savings from Turbo Boost. The reason can be attributed to the memory bounded nature of these workloads that does not scale well with the CPU frequency, thus it is a waste of power consumption for the CPU frequency staying at the highest frequency during Turbo stage.

However, beyond our expectation, the results illustrated in Fig 5(a) seem that Turbo Boost is not an effective way to execute the workload in terms of performance delivered per watt, which are intuitively conflicting with the energy savings shown in Fig 5(d). After verifying the experimental data, the confusing results can be attributed to the performance metric adopted to evaluate the energy efficiency of Turbo Boost. Although IPC is a good performance indictor for applications when the CPU frequency remains constant, it is incapable to depict the impact of frequency speeding on energy efficiency. As known, once the application and the execution environment is determined, the IPC of a particular application stays almost unchanged regardless of the CPU frequency, which is in accordance with our experimental data. The only thing affected by CPU frequency is the actual time consumed by each CPU cycle. The higher the CPU frequency is, the shorter time each CPU cycle takes. Therefore, when the Turbo Boost is enabled, the power consumption increases and the total execution time decreases apparently, whereas the IPC of the application is hardly affected. This explains the discrepancy between energy efficiency and energy consumption results in Fig 5(a) and 5(d).

**Fig 6:**
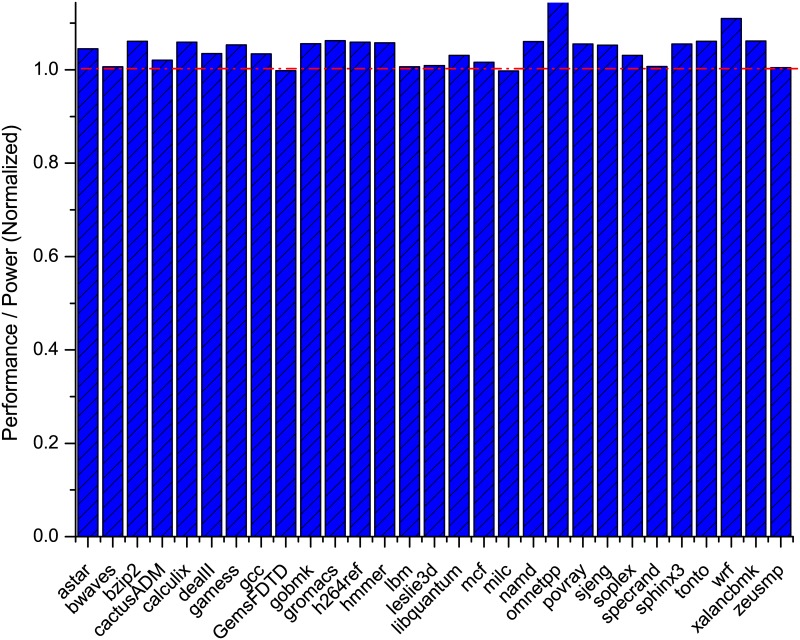
Energy efficiency of Turbo Boost with SPECCPU workloads using IPS as performance metric.

To ease the understanding of the energy efficiency results, we propose to utilize the metric of Instructions Per Second (IPS) as the performance indicator for evaluating the energy efficiency of Turbo Boost. IPS can be calculated by multiplying IPC with CPU frequency, and the energy efficiency is eventually described by Eq 1. The energy efficiency results of SPECCPU workloads with Turbo Boost enabled are regenerated in Fig 6, which are now in consistent with the energy savings revealed in Fig 5(d). As shown in Fig 6, the energy efficiency for most of the SPECCPU workloads has been improved by 4.3% on average with Turbo Boost enabled. The most energy efficiency improvement is achieved by 13% with *omnetpp*, whereas for the worst cases (e.g., *GemsFDTD* and *milc*) the energy efficiency dose not degrade if not at all compared to Turbo Boost turned off.

$$\begin{array}{c} EnergyEfficiency=\frac{IPS}{Power}=\frac{Instructions*Frequency}{Cycles*Power} \end{array}$$ (1)

**Insight-3 (Turbo Boost)** *Turbo Boost is effective to boost the execution and further preserve the energy for workloads from system perspective. With Turbo Boost enabled, the reduction in execution time offsets the increase in power consumption, thus the energy efficiency of the workloads is improved uniformly. For a long term goal of energy saving, it is always recommended to leave Turbo Boost turned on. However, for instantaneous power consumption, enabling Turbo Boost generates significant power surge (e.g., 20.6% for SPECCPU workloads), which may constrain its adoption on systems with tight power budget. It is worth mention that since Turbo Boost adjusts the CPU frequency when effective, the evaluation metric (e.g., IPS) should take into account the frequency adjustment in order to accurately measure the energy efficiency.*

## Conclusion and future work

In this paper, we first highlighted the motivation to evaluate the architecture designs from energy efficiency perspective. We illustrated the energy proportionality gap of current server system with quantitive analysis in order to justify the necessity of our study. We also described the energy measurement capability of RAPL interfaces which were applied in our evaluation. The deviation of the time interval between each RAPL energy update was as well identified through statistical analysis. With representative benchmark suites ranging from serial to parallel workloads, we characterized the architecture designs such as NUMA, SMT and Turbo Boost in terms of power consumption, energy consumption as well as energy efficiency. Accompanied with the results, we presented comprehensive analysis with insights to guide energy efficient system designs in the future.

Since service based application gradually becomes dominant, especially in cloud computing environment, and stands for the future trend of application ecosystem, we would like to extend our evaluation to incorporate cloud style benchmarks such as cloudsuite [39] for the future work. And we are eager to find out more interesting results on how the existing architecture designs interact with these emerging applications.

## Supporting information

S1 Table NUMA experiment data including energy efficiency, average power consumption, energy consumption as well as energy ratio across RAPL domains (PP0/UNCORE/DRAM). (PDF)

S2 Table SMT experiment data including energy efficiency, average power consumption, energy consumption as well as energy ratio across RAPL domains (PP0/UNCORE/DRAM). (PDF)

S3 Table Turbo Boost experiment data including energy efficiency, average power consumption, energy consumption as well as energy ratio across RAPL domains (PP0/UNCORE/DRAM). (PDF)
