# Supplementary material for: Evaluating architecture impact on system energy efficiency
Source: PLoS One. 2017 Nov 21;12(11):e0188428. doi: 10.1371/journal.pone.0188428 (PMC5697812; doi:10.1371/journal.pone.0188428)
Supplement: S1 Table — (PDF) [file pone.0188428.s001.pdf]

Performace/Power (Normalized)					
	CLASS=S	CLASS=W	CLASS=A	CLASS=B	CLASS=C
ep.4	0.98675	0.97782	0.96816	0.96432	0.96784
mg.4	0.95642	0.75342	0.66369	0.67534	0.63904
cg.4	0.9693	0.95777	0.61577	0.62195	0.72208
ft.4	0.93763	0.83458	0.78957	0.81462	0.82451
is.4	0.94144	0.88108	0.76829	0.77766	0.78638
lu.4	0.99501	0.96903	0.8663	0.85581	0.71906
sp.4	0.7619	0.81348	0.66493	0.62674	0.61673
bt.4	0.97609	0.97311	0.7849	0.8003	0.80152

Energy Rato (PP0/UNCORE/DRAM) - Baseline					
	CLASS=S	CLASS=W	CLASS=A	CLASS=B	CLASS=C
ep.4	0.23112/0.65503/0.11385	0.2984/0.59864/0.10296	0.49392/0.43303/0.07305	0.55355/0.38248/0.06397	0.57216/0.36679/0.06106
mg.4	0.1466/0.72573/0.12767	0.23037/0.66351/0.10611	0.44133/0.50884/0.04983	0.54922/0.42831/0.02247	0.60735/0.38975/0.0029
cg.4	0.16043/0.71375/0.12582	0.2259/0.66096/0.11315	0.37293/0.55951/0.06756	0.59262/0.37878/0.0286	0.59439/0.37461/0.03099
ft.4	0.18134/0.69659/0.12207	0.21979/0.66829/0.11192	0.51712/0.43464/0.04824	0.61381/0.35684/0.02935	0.62762/0.34299/0.02939
is.4	0.14693/0.72753/0.12553	0.19327/0.68939/0.11734	0.37018/0.54636/0.08346	0.49335/0.44698/0.05968	0.5481/0.40238/0.04952
lu.4	0.15498/0.71769/0.12732	0.52422/0.40862/0.06716	0.59442/0.36518/0.04039	0.6065/0.3594/0.0341	0.60011/0.39529/0.0046
sp.4	0.16102/0.71415/0.12483	0.52699/0.42619/0.04682	0.57829/0.41346/0.00826	0.57926/0.41516/0.00558	0.5821/0.41069/0.00722
bt.4	0.16881/0.70688/0.12431	0.44783/0.47276/0.07941	0.60705/0.3585/0.03445	0.61889/0.34933/0.03178	0.61922/0.34858/0.0322

Energy Rato (PP0/UNCORE/DRAM) - NUMA					
	CLASS=S	CLASS=W	CLASS=A	CLASS=B	CLASS=C
ep.4	0.23112/0.65503/0.11385	0.2885/0.60671/0.10479	0.49407/0.43281/0.07312	0.55512/0.38148/0.0634	0.57231/0.36687/0.06082
mg.4	0.1466/0.72573/0.12767	0.25038/0.63889/0.11074	0.46853/0.45468/0.07678	0.56346/0.3748/0.06174	0.60917/0.33621/0.05463
cg.4	0.16043/0.71375/0.12582	0.22811/0.65844/0.11346	0.40966/0.50435/0.08599	0.58648/0.3552/0.05831	0.58781/0.35405/0.05814
ft.4	0.18134/0.69659/0.12207	0.23465/0.65236/0.11299	0.52824/0.40432/0.06743	0.61458/0.33103/0.05439	0.62605/0.32123/0.05272
is.4	0.14693/0.72753/0.12553	0.19267/0.68798/0.11935	0.39075/0.5208/0.08845	0.50992/0.41888/0.0712	0.55253/0.38397/0.0635
lu.4	0.15498/0.71769/0.12732	0.5238/0.40751/0.06869	0.59649/0.34642/0.05709	0.60785/0.33706/0.05509	0.60406/0.34073/0.05521
sp.4	0.16102/0.71415/0.12483	0.53571/0.3983/0.06599	0.58432/0.35764/0.05804	0.5867/0.35549/0.0578	0.58748/0.35477/0.05775
bt.4	0.16881/0.70688/0.12431	0.44776/0.4719/0.08034	0.60328/0.34067/0.05605	0.61417/0.33128/0.05454	0.61447/0.33105/0.05448

Average Power (Normalized)					
	CLASS=S	CLASS=W	CLASS=A	CLASS=B	CLASS=C
ep.4	0.99411	0.99527	1.03217	1.03746	1.03377
mg.4	0.98605	1.05337	1.09508	1.06542	1.00895
cg.4	1.00621	1.02157	1.0996	1.01895	1.01689
ft.4	1.03167	1.04386	1.06348	1.03447	1.02723
is.4	1.03273	1.01714	1.06999	1.05836	1.04637
lu.4	0.99242	1.02673	1.03763	1.03958	1.03609
sp.4	0.99097	1.05731	1.03455	1.0232	1.01917
bt.4	0.99897	1.02705	1.02296	1.01964	1.01801

Energy Consumption (Normalized)					
	CLASS=S	CLASS=W	CLASS=A	CLASS=B	CLASS=C
ep.4	0.97701	0.96486	1.0016	1.00502	1.00294
mg.4	0.96532	1.05089	1.18628	1.24751	1.38879
cg.4	1.00423	1.0008	1.19426	1.3081	1.29554
ft.4	1.0139	1.03001	1.11797	1.13267	1.13674
is.4	1.01203	1.01208	1.11708	1.17979	1.20911
lu.4	0.98822	1.00203	1.09442	1.10151	1.26573
sp.4	0.95143	1.12195	1.34209	1.40429	1.40486
bt.4	0.98821	1.00222	1.19448	1.17332	1.17925
