# Supplementary material for: Evaluating architecture impact on system energy efficiency
Source: PLoS One. 2017 Nov 21;12(11):e0188428. doi: 10.1371/journal.pone.0188428 (PMC5697812; doi:10.1371/journal.pone.0188428)
Supplement: S2 Table — (PDF) [file pone.0188428.s002.pdf]

Performace/Power (Normalized)				
	Small	Medium	Large	Native
blackscholes	0.92147	1.12771	1.1415	1.16579
bodytrack	1.02924	1.07684	1.09749	1.10057
ferret	1.00356	1.02052	1.11992	1.26772
freqmine	0.99433	0.99447	1.00012	0.99755
raytrace	1.00448	1.00488	1.00775	1.08392
swaptions	1.02822	0.74762	1.13052	1.00099
vips	1.03382	1.06934	1.07527	1.06732
x264	0.94043	1.01158	0.95692	1.11605

Energy Rato (PP0/UNCORE/DRAM) - Baseline				
	Small	Medium	Large	Native
blackscholes	0.30128/0.65064/0.04808	0.54502/0.42654/0.02844	0.55397/0.41811/0.02792	0.55627/0.41611/0.02762
bodytrack	0.52857/0.44286/0.02857	0.58205/0.39189/0.02606	0.60342/0.37187/0.02471	0.60856/0.36704/0.02441
ferret	0.49202/0.47606/0.03191	0.57787/0.39581/0.02632	0.60565/0.37046/0.02388	0.62576/0.35132/0.02292
freqmine	0.38857/0.57315/0.03828	0.3936/0.56857/0.03783	0.36261/0.59761/0.03979	0.3935/0.56865/0.03785
raytrace	0.39696/0.56529/0.03774	0.40457/0.55825/0.03719	0.41739/0.54616/0.03646	0.49934/0.46948/0.03118
swaptions	0.46791/0.49733/0.03476	0.57805/0.39593/0.02602	0.58314/0.39088/0.02598	0.61944/0.35677/0.02379
vips	0.55112/0.42145/0.02743	0.61843/0.35736/0.0242	0.62703/0.34961/0.02336	0.63342/0.34378/0.0228
x264	0.48947/0.47895/0.03158	0.61682/0.35903/0.02414	0.64162/0.33614/0.02225	0.65548/0.3238/0.02072

Energy Rato (PP0/UNCORE/DRAM) - SMT				
	Small	Medium	Large	Native
blackscholes	0.30921/0.64803/0.04276	0.56684/0.40642/0.02674	0.56697/0.40609/0.02693	0.5681/0.40505/0.02685
bodytrack	0.5358/0.43457/0.02963	0.59566/0.37849/0.02585	0.61698/0.35896/0.02406	0.6244/0.35221/0.02339
ferret	0.49796/0.47075/0.03129	0.57364/0.39978/0.02658	0.61546/0.36145/0.02309	0.66209/0.31748/0.02042
freqmine	0.3871/0.57492/0.03798	0.39383/0.56836/0.03781	0.37751/0.58384/0.03865	0.39315/0.56898/0.03787
raytrace	0.39877/0.56371/0.03752	0.40437/0.55853/0.0371	0.41663/0.54692/0.03646	0.49952/0.46932/0.03116
swaptions	0.4779/0.48895/0.03315	0.50465/0.46492/0.03043	0.6077/0.36782/0.02448	0.61245/0.36335/0.0242
vips	0.56722/0.40589/0.02689	0.64152/0.33645/0.02203	0.65175/0.32793/0.02032	0.65853/0.32124/0.02023
x264	0.48775/0.48039/0.03186	0.62022/0.35589/0.02389	0.63095/0.34627/0.02277	0.67863/0.30147/0.01989

Average Power (Normalized)				
	Small	Medium	Large	Native
blackscholes	0.78358	0.63691	0.62636	0.61512
bodytrack	0.62178	0.58499	0.56566	0.56808
ferret	0.87451	0.83463	0.73053	0.56227
freqmine	0.9997	0.99906	1.0163	0.99878
raytrace	0.98095	0.96147	0.8876	0.67782
swaptions	0.7037	0.95855	0.58914	0.65375
vips	0.57972	0.55108	0.54562	0.54387
x264	0.92407	0.8586	0.84686	0.57619

Energy Consumption (Normalized)				
	Small	Medium	Large	Native
blackscholes	0.97436	0.88626	0.87531	0.85899
bodytrack	0.96429	0.9334	0.91275	0.9097
ferret	0.97739	0.96992	0.89715	0.7888
freqmine	1.00787	1.00852	1.00804	1.00251
raytrace	0.99789	0.99771	0.99215	0.92326
swaptions	0.96791	1.33824	0.88453	0.99959
vips	0.97382	0.93512	0.93299	0.93913
x264	1.07368	0.97819	1.04829	0.89779
