# Supplementary material for: Evaluating architecture impact on system energy efficiency
Source: PLoS One. 2017 Nov 21;12(11):e0188428. doi: 10.1371/journal.pone.0188428 (PMC5697812; doi:10.1371/journal.pone.0188428)
Supplement: S3 Table — (PDF) [file pone.0188428.s003.pdf]

	Performace/Power (Normalized)	Average Power (Normalized)	Energy Consumption (Normalized)	Energy Rato (PP0/UNCORE/DRAM)	
				Baseline	Turbo Boost
astar	0.83583	1.17512	0.95746	0.39168/0.57035/0.03797	0.48131/0.48646/0.03223
bwaves	0.80526	1.17432	0.99493	0.39653/0.56704/0.03642	0.48435/0.48481/0.03084
bzip2	0.84902	1.17485	0.94249	0.39218/0.5699/0.03792	0.48197/0.48578/0.03225
cactusADM	0.81644	1.1775	0.98025	0.40413/0.55893/0.03694	0.49247/0.47623/0.0313
calculix	0.84733	1.17825	0.9444	0.39323/0.56892/0.03786	0.48377/0.48414/0.03209
dealII	0.8277	1.17801	0.96711	0.39584/0.56685/0.03731	0.48562/0.48284/0.03154
gamess	0.50091	1.99492	0.94578	0.39833/0.56399/0.03768	0.48946/0.47873/0.0318
gcc	0.82747	1.17524	0.96707	0.39726/0.56544/0.0373	0.4859/0.48242/0.03168
GemsFDTD	0.7664	1.19027	1.04598	0.3995/0.56483/0.03567	0.48119/0.47733/0.04148
gobmk	0.84498	1.17742	0.94727	0.39409/0.56751/0.0384	0.48376/0.4835/0.03274
gromacs	0.84998	1.17793	0.94151	0.39164/0.57022/0.03814	0.48237/0.48539/0.03224
h264ref	0.84718	1.18199	0.94483	0.40087/0.56172/0.03741	0.49188/0.47649/0.03163
hmmer	0.84627	1.18051	0.94568	0.39672/0.56552/0.03775	0.48778/0.4803/0.03192
lbm	0.76607	1.17161	1.04566	0.39258/0.57098/0.03644	0.4799/0.48904/0.03107
leslie3d	0.87128	1.07794	1.00515	0.39639/0.56786/0.03575	0.48466/0.48483/0.03051
libquantum	0.82465	1.19574	0.97242	0.3933/0.56969/0.03701	0.47461/0.48553/0.03986
mcf	0.81273	1.17718	0.98605	0.39657/0.56659/0.03683	0.4852/0.48352/0.03127
milc	0.70266	1.28514	1.04335	0.3945/0.56876/0.03674	0.48198/0.48659/0.03143
namd	0.84824	1.17756	0.94346	0.39328/0.56883/0.03789	0.48349/0.48436/0.03215
omnetpp	0.97581	1.18304	0.82068	0.39048/0.57151/0.03802	0.48091/0.48764/0.03144
povray	0.84423	1.18309	0.94808	0.40424/0.55844/0.03732	0.49539/0.47318/0.03143
sjeng	0.84208	1.17786	0.95045	0.39133/0.5707/0.03798	0.48181/0.48597/0.03222
soplex	0.82451	1.1771	0.97201	0.39367/0.56931/0.03702	0.4821/0.48675/0.03115
specrand	0.87755	1.14283	0.99452	0.38904/0.5726/0.03836	0.44353/0.52342/0.03306
sphinx3	0.84445	1.18301	0.94779	0.40385/0.55889/0.03726	0.49473/0.47381/0.03146
tonto	0.84883	1.18071	0.94287	0.39891/0.56353/0.03756	0.48981/0.47843/0.03176
wrf	0.88802	1.18037	0.90166	0.39193/0.57019/0.03788	0.48138/0.4868/0.03182
xalancbmk	0.84929	1.17901	0.94237	0.39559/0.56677/0.03765	0.48529/0.48286/0.03185
zeusmp	0.80352	1.17709	0.99627	0.3946/0.56808/0.03732	0.48387/0.4845/0.03163
